# Modulation of Macrophages Using Nanoformulations with Curcumin to Treat Inflammatory Diseases: A Concise Review

**DOI:** 10.3390/pharmaceutics14102239

**Published:** 2022-10-20

**Authors:** Huxiao Sun, Mengsi Zhan, Serge Mignani, Dzmitry Shcharbin, Jean-Pierre Majoral, João Rodrigues, Xiangyang Shi, Mingwu Shen

**Affiliations:** 1 State Key Laboratory for Modification of Chemical Fibers and Polymer Materials, Shanghai Engineering Research Center of Nano-Biomaterials and Regenerative Medicine, College of Biological Science and Medical Engineering, Donghua University, Shanghai 201620, China; 2 CQM—Centro de Química da Madeira, MMRG, Universidade da Madeira, Campus Universitário da Penteada, 9020-105 Funchal, Portugal; 3 Laboratoire de Chimie et de Biochimie Pharmacologiques et Toxicologique, Université Paris Descartes, PRES Sorbonne Paris Cité, CNRS UMR 860, 45, rue des Saints Peres, 75006 Paris, France; 4 Institute of Biophysics and Cell Engineering of NASB, Akademicheskaya 27, 220072 Minsk, Belarus; 5 Laboratoire de Chimie de Coordination du CNRS, 205 Route de Narbonne, CEDEX 4, 31077 Toulouse, France; 6 Laboratoire de Chimie de Coordination du CNRS, Université Toulouse, 31077 Toulouse, France

**Keywords:** curcumin, nanoformulations, bioavailability, drug delivery, inflammation

## Abstract

Curcumin (Cur), a traditional Chinese medicine extracted from natural plant rhizomes, has become a candidate drug for the treatment of diseases due to its anti-inflammatory, anticancer, antioxidant, and antibacterial activities. However, the poor water solubility and low bioavailability of Cur limit its therapeutic effects for clinical applications. A variety of nanocarriers have been successfully developed to improve the water solubility, in vivo distribution, and pharmacokinetics of Cur, as well as to enhance the ability of Cur to polarize macrophages and relieve macrophage oxidative stress or anti-apoptosis, thus accelerating the therapeutic effects of Cur on inflammatory diseases. Herein, we review the design and development of diverse Cur nanoformulations in recent years and introduce the biomedical applications and potential therapeutic mechanisms of Cur nanoformulations in common inflammatory diseases, such as arthritis, neurodegenerative diseases, respiratory diseases, and ulcerative colitis, by regulating macrophage behaviors. Finally, the perspectives of the design and preparation of future nanocarriers aimed at efficiently exerting the biological activity of Cur are briefly discussed.

## 1. Introduction

Inflammation is the body’s response triggered by certain stimuli, such as viral or bacterial infections, mechanical damage, and excessive immune responses [1]. The purpose of the inflammation response is to remove stimulating factors and accelerate tissue repair, but excessive inflammation response may cause tissue damage, organ dysfunction, and even life threatening [2]. If the excessive inflammation response cannot be controlled over time, the occurrence of infectious or systemic diseases, including systemic lupus erythematosus, rheumatoid arthritis (RA), atherosclerosis, and pneumonia, will be caused [3]. In the inflammatory microenvironment, immune cells, such as neutrophils, monocytes, macrophages, and lymphocytes, infiltrate inflammatory tissues to varying degrees [4]. Macrophages, as the innate immune system, have a wide range of essential functions in the initiation and execution of inflammatory responses [5,6]. The balance between M1 and M2 macrophages can be disrupted when macrophages are activated, thus secreting a large number of inflammatory mediators, including tumor necrosis factor-α (TNF-α), interleukin (IL)-1β and IL-6 [7]. These pro-inflammatory cytokines contribute to the occurrence and development of inflammatory diseases.

In addition, reactive oxygen species (ROS), such as hydrogen peroxide (H_2_O_2_), superoxide radical anion (O_2_•−), and hydroxyl radical (•OH), are homeostasis in normal tissues and play important roles in maintaining cell metabolism, proliferation, and differentiation [8]. However, ROS levels in the inflammatory microenvironment can be abnormally elevated, triggering excessive oxidative stress in cells, thereby leading to DNA damage, cellular dysfunction, and further aggravated tissue damage [1,9]. Therefore, taking measures such as reducing pro-inflammatory cytokines, eliminating ROS, and controlling the infiltration of inflammatory cells are major strategies to improve the treatment efficiency of inflammatory diseases [10,11,12,13].

Based on the above treatment strategies, many anti-inflammatory drugs have been developed to reduce the inflammatory response, including non-steroidal anti-inflammatory drugs (NSAIDs) [14] and glucocorticoids [15,16]. However, high doses of these drugs can cause adverse effects to the body, such as liver damage, pulmonary fibrosis, and gastrointestinal discomfort [17]. Finding safe and effective anti-inflammatory drugs is still the top priority in eliminating inflammatory diseases. Curcumin (Cur), 1,7-bis(4-hydroxy-3-methoxyphenyl)-1,6-heptadiene-3,5-dione, is a polyphenolic compound derived from the rhizome of the herb *Curcuma longa* [18], and exhibits a pH-dependent keto–enol tautomerism. In an acidic or neutral solution, the keto form of Cur is predominant, while in an alkaline medium, the enol form of Cur becomes predominant [19]. Cur has received extensive attention as a Chinese herbal medicine with outstanding physiological activity and remarkable biosafety. Meanwhile, Cur shows excellent antioxidant and anti-inflammatory performance. On one hand, the phenolic hydroxyl group of Cur endows it with excellent antioxidant ability [20]; on the other hand, Cur can reduce ROS levels by decreasing the expression of nicotinamide adenine dinucleotide phosphate (NADPH) oxidase and nitric oxide synthase (NOS) and enhancing antioxidant enzyme activity [21], and inhibit the production of inflammatory cytokines by regulating mitogen-activated protein kinase (MAPK), janus kinase/signal transducer and activator of transcription (JAK/STAT) and nuclear factor kappa-B (NF-κB) signaling pathways, thereby exerting an anti-inflammatory effect [22,23]. Hence, Cur, with its anti-inflammatory and antioxidant properties, has become a promising candidate drug for the treatment of inflammatory diseases. Nonetheless, the therapeutic activity of Cur is limited due to its poor water solubility, fast biological metabolism, and poor bioavailability [24,25,26,27]. The dose or frequency of administration must be increased to achieve the desired therapeutic effects, which in turn cause adverse reactions to the body. Clinical studies have shown that Cur at doses ranging from 0.9 to 3.6 g per day for 1–4 months has some adverse effects, including nausea and diarrhea, and can cause an increase in serum alkaline phosphatase and lactate dehydrogenase [28]. In addition, the administration of a high dose of Cur has the potential to affect whole-body iron metabolism, particularly in people with suboptimal iron status [29]. Therefore, improving the poor bioavailability of Cur is an important step in amplifying its application value.

**Figure 1:**
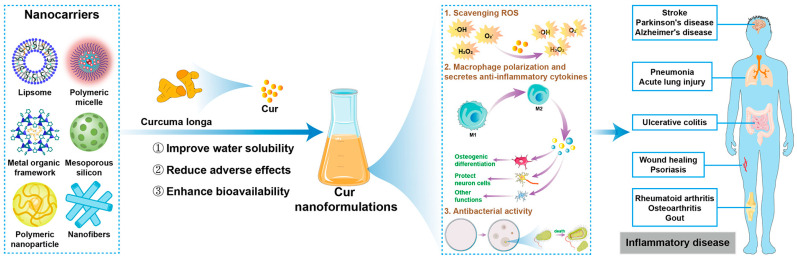
Schematic diagram of the preparation of Cur nanoformulations and their therapeutic application in inflammatory diseases.

With the development of nanotechnology, a series of advances in Cur-loaded nanocarriers have been developed to solve this problem (Figure 1). Nanocarriers, such as liposomes, polymeric micelles, metal organic frameworks (MOFs), inorganic nanocarriers, polymeric nanoparticles (NPs), proteins, nanofibers, etc., have been designed to enhance the bioavailability and biological functions of Cur (representative samples are shown in Table 1). In order to improve the distribution of Cur in vivo, realize the controllable release of Cur at the lesion site, and achieve successful precise treatment of diseases, various strategies such as size control, surface functionalization, intelligent stimuli-responsiveness design, and biomimetic coating of nanocarriers have been developed [30,31,32,33]. As a promising drug, Cur has been evaluated in various settings, with positive results in the treatment of inflammatory diseases using diverse types of nanoformulations [34]. Therefore, this review aims to introduce the miscellaneous types of Cur nanoformulations, summarize the recent progress of Cur nanoformulations in inflammatory disease treatment, and discuss the perspectives in the future design and biological applications of Cur nanoformulations.

## 2. Curcumin Nanoformulations

In this section, we mainly discuss the efficient delivery of Cur using various types of nanomaterials, including liposomes, polymeric micelles, MOFs, inorganic nanocarriers, polymeric NPs, and other nanomaterials. These nanomaterials amplify the biological activity and therapeutic effect of Cur by improving the water solubility, stability, and bioavailability of Cur, as well as achieving controlled release of Cur.

### 2.1. Lipid-Based Nanoparticles

Liposomes can be used to efficiently encapsulate hydrophilic and hydrophobic drugs due to their unique bilayer phospholipid structure with hydrophilic interiors [19]. Liposomes prepared with different components have also been developed as carriers for Cur delivery. For instance, Tai et al. [35] constructed Cur-loaded liposomes via a thin-film hydration method using soybean phospholipid (SPC), hydrogenated soybean phospholipid (HSPC), cholesterol, and Cur. The size, encapsulation efficiency, and stability of the Cur-loaded liposomes varied with the ratio of SPC and HSPC in liposomes. Compared with pure SPC liposomes or pure HSPC liposomes, the mixed phospholipid liposomes composed of SPC/HSPC at a ratio of 5:5 not only retain the physical stability of SPC liposomes but also maintain the characteristics of HSPC liposomes, such as high encapsulation efficiency and sustained release properties. In addition to changing the type and ratio of phospholipids in liposomes, the Cur encapsulation efficiency and stability of liposomes can be improved by adjusting the environment in the inner chamber of the liposomes. For example, using poloxamer 188 (F68) phosphate buffer with different pH values as the aqueous phase and the phospholipids/cholesterol as the organic phases, liposomes (CURC-LP) with different pH values (pH = 2.5, 5.0, or 7.0) can be formed to encapsulate Cur in their inner cavities [36]. In this study, the Cur encapsulation efficiency of the constructed liposomes was 73.7 ± 1.6% when the pH of the inner chamber was 2.5, which was higher than that of CURC-LP (pH 5.0, 40 ± 2.2%) and CURC-LP (pH 7.4, 64 ± 1%). The particle size of CURC-LP did not significantly change with the pH value of the inner cavity of liposomes; however, more Cur was retained in CURC-LP (pH = 2.5) within 24 h than in CURC-LP (pH = 5.0) and CURC-LP (pH = 7.4) in a physiological environment (50%, pH 7.4, fetal bovine serum). It can be seen that creating an acidic environment in the inner chamber of the liposomes is an effective strategy to improve the encapsulation efficiency of Cur and to facilitate the stability of Cur-loaded liposomes.

To solve the problem of non-specific targeting of Cur-loaded liposomes, Qi et al. [30] prepared a multifunctional liposome (CAPS-Cur/GA&Gal-Lip) loaded with Cur and capsaicin (CAPS) inside, and modified with glycyrrhetinic acid (GA) and galactose (Gal) on their surface for targeted therapy of liver cancer. Compared with non-targeting and single-targeting liposomes, the prepared CAPS-Cur/GA&Gal-Lip displayed good liver cancer-targeting specificity due to the modification of double-targeting ligands. In addition, Cur-loaded liposomes can be coated with polymer compounds through cross-linking to form injectable hydrogels, which further improves the in vitro and in vivo stability of Cur-loaded liposomes and delays their release [47,48]. This is also a promising strategy for further improving the application of Cur-loaded liposomes in drug delivery and tissue engineering. Overall, the strategies of changing the phospholipid composition, inner cavity environment, or surface modification have great potential to overcome the shortcomings of traditional liposomes with poor stability, non-targeting specificity, and low bioavailability [19,49].

In addition to liposomes, other lipid-based NPs, such as solid lipid NPs (SLNs), nanostructured lipid carriers (NLCs), and nanoemulsions (NEs), have also become the delivery systems of hydrophobic drugs due to their unique properties [50]. Ganesan et al. [51] used high shear homogenization and ultrasonication techniques to prepare Cur-loaded SLNs (SLCN), which could inhibit the lipopolysaccharide (LPS)-activated inflammatory response in BV2 cells more effectively than free Cur due to the continuous release and improved bioavailability of Cur. In another study, Malvajerd et al. [52] encapsulated Cur in SLNs and NLCs (Cur-SLNs and Cur-NLCs) to achieve Cur delivery to brain tissue. They found that the Cur-SLNs and Cur-NLCs all showed a certain sustained release performance compared to the explosive release of free Cur, and Cur-NLCs exhibited better storage stability than Cur-SLNs, resulting in a 4-fold enhanced uptake of Cur by brain tissue and having a great therapeutic potential for central nervous system diseases. In addition, transferrin-functionalized SLNs and NLCs have also been developed to address the obstacle of Cur crossing the blood–brain barrier (BBB) [53]. NEs are homogeneous dispersion systems with particle sizes of 1 to 100 nm formed by water, oil, surfactants, and co-surfactants, which have also been designed to improve Cur performance. Chen et al. [54] achieved controllable physicochemical properties and stability of Cur-loaded NEs by adjusting the oil type (medium chain triglyceride oil and canola oil), emulsifier type (Tween-80 and lecithin), and surfactant/oil ratio. Li et al. [55] prepared chitosan-coated Cur-loaded NEs with 95% encapsulation efficiency, and the functionalization of chitosan effectively inhibited the degradation of Cur by heat and ultraviolet irradiation and improved the stability of Cur-loaded NEs.

### 2.2. Polymeric Micelles

Polymeric micelles with a “core-shell” structure are formed from amphiphilic block polymers in an aqueous solution by self-assembly. The shell of the micelles is composed of hydrophilic blocks, which endow the micelles with protection from capture by the reticuloendothelial system (RES) organs and prolonged blood circulation time in vivo [56]. The hydrophobic core of the micelles can be used as a reservoir to incorporate hydrophobic drugs [57] to solubilize drugs with poor water solubility, showing great potential in drug delivery systems. Sohail et al. [58] designed polymeric micelles (CPM-DD) composed of amphiphilic diblock copolymers (mPEG-PLA) to co-encapsulate dimethoxycurcumin (DiMC) and doxorubicin (DOX). The cumulative release rate of DOX in CPM-DD was as high as 84% within 72 h, while DiMC exhibited a sustained release profile, reaching about 40% release within 72 h, indicating strong attachments of DiMC to the constructed micelles. Recently, stimuli-responsive components have been introduced into micelles to achieve the release of Cur under specific microenvironments, thus enhancing drug efficacy and reducing its side effects. For instance, Li et al. [37] synthesized glutathione (GSH)-responsive organosilicon hybrid nanomicelles ((Gen + Cur)@FOS) using polyethyleneoxide 100-polypropyleneoxide 65-polyethyleneoxide 100 (PEO_100_-PPO_65_-PEO_100_) triblock copolymers to load both Cur and genistein. The (Gen + Cur)@FOS with a particle size of about 13.8 nm showed good stability, which was demonstrated by negligible change in their hydrodynamic size within one week. Compared with the cumulative release of Cur in a GSH-free environment of about 18% at 36 h, the release of Cur was as high as 72.4% under a GSH solution at a concentration of 10 mM. This indicated the excellent redox-responsive drug release property of the (Gen + Cur)@FOS. In addition to the redox-responsive polymeric micelles, various pH-responsive and hypoxia-responsive Cur-loaded polymeric micelles have also been developed for controlled release of Cur [31,59,60].

Notably, besides blending Cur with amphiphilic block copolymers and self-assembling it into Cur-loaded polymer micelles, covalently linking Cur to the hydrophilic polymer backbone to form amphiphilic block copolymers has also been proposed to prepare Cur-loaded polymeric micelles [61,62,63]. Recently, amphiphilic dendron [22,64] and amphiphilic polypeptide [65] have been designed to form micelles for Cur delivery. In a recent example reported by Li et al. [22], a third-generation amphiphilic phosphorus dendron with excellent intrinsic anti-inflammatory activity was used to form Cur-loaded micelles (C11G3-TBP@Cur) for combination anti-inflammatory and anti-oxidative treatment of acute lung injury. The aromatic backbone structure of phosphorus dendrons brings the nanomicelles with great rigidity and stability, and only 20% of Cur can be released from the C11G3-TBP@Cur complexes within 9 days, thus avoiding the burst release of Cur. In addition, various targeting molecules can be modified on the surface of polymeric micelles, including mitochondrial targeting agent [66], GA [67], and peptide [68], etc., so that Cur-loaded polymeric micelles can be precisely localized to the disease area to reduce the toxicity of drugs to normal tissues.

### 2.3. Metal–Organic Frameworks

MOFs, porous coordination polymers, are formed by the self-assembly of metal ions and organic ligands. The void space of MOFs endows them with a high specific surface area, which is beneficial for drug encapsulation and controlled release [69]. Since MOFs were explored as drug delivery carriers in 2006, MOFs have achieved exceptionally brilliant results in the biomedical field and have been regarded as an important candidate in the development of drug delivery system [70]. Zeolitic imidazole frameworks (ZIFs) with pH-responsiveness, as an important example of many types of MOFs, have been constructed from zinc ions and dimethylimidazole and developed for the controlled release of Cur. For instance, Zheng et al. [38] prepared Cur-loaded nanoscale ZIF-8 (CCM@NZIF-8) via a simple nanoprecipitation method. The CCM@NZIF-8 NPs with high encapsulation efficiency (88.2%) and acid-responsive drug release property were more toxic to HeLa cells than free Cur because of the enhanced cellular uptake of Cur after being encapsulated.

**Figure 2:**
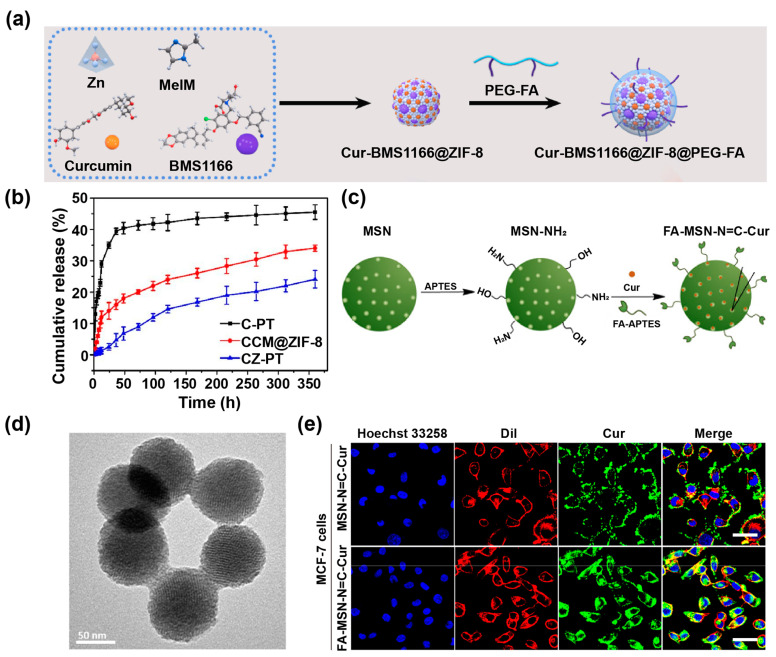
(**a**) Schematic diagram of the preparation of Cur-BMS1166@ZIF-8@PEG-FA. Reproduced with permission from [39], Copyright 2022, Elsevier. (**b**) Cur release from CCM@ZIF-8, C-PT and CZ-PT under neutral conditions. Reproduced with permission from [72], Copyright 2020, Elsevier. (**c**) Schematic diagram of the preparation of FA-MSN-N=C-Cur. (**d**) TEM image of FA-MSN-N=C-Cur. (**e**) The cellular uptake of MCF-7 cells after incubation with MSN-N=C-Cur and FA-MSN-N=C-Cur for 6 h, as observed by confocal laser scanning microscope (CLSM) (Scale bars = 50 μm). Reproduced with permission from [42], Copyright 2018, Elsevier.

However, MOFs have some shortcomings in the process of disease treatment because of their strong serum protein adsorption capacity, and they are easily cleared by RES organs to reduce their bioavailability. In response to these problems, researchers have adopted strategies of polymer modification, targeting molecule functionalization, and biomimetic cell membrane coating to enhance the stability and targeting specificity of MOFs. For example, Zhang et al. [40] coated polydopamine on the surface of Cur-loaded ZIF, which endowed it with systemic circulation stability and photothermal conversion performance in vivo. In another study, Ge et al. [39] coated the surface of Cur-loaded ZIF (Cur-BMS1166@ZIF-8) with folic acid (FA)-grafted polyethylene glycol (PEG-FA) to target osteosarcoma for improved Cur delivery (Figure 2a). In addition, Cur-loaded ZIF can be coated with tumor cell membranes to achieve homologous tumor targeting and drug delivery [71]. Furthermore, the strategy of encapsulating Cur-loaded ZIF as guests in other host materials to form composites has been used to enhance the performance of MOFs and expand the delivery application field of Cur. Wang et al. [72] encapsulated Cur-loaded ZIF (CCM@ZIF-8) into poly (L-lactic acid) nanofiber scaffolds by electrospinning methods to form composite scaffolds (CZ-PT) for the treatment of diabetic wound healing. Compared with CCM@ZIF-8, CZ-PT further delayed the release of Cur, with a release time prolonged up to 15 days (Figure 2b), ensuring that Cur could be released at the appropriate rate and concentration throughout the treatment period to achieve the desired therapeutic effects.

### 2.4. Inorganic Nanomaterials

In recent years, inorganic nanomaterials, including inorganic metal-based materials and inorganic non-metal-based materials, have received widespread attention for the construction of drug delivery systems. Inorganic metal-based nanomaterials, represented by layered double hydroxide (LDH) with a brucite-like layered two-dimensional structure, are composed of hydroxides of divalent and trivalent metal cations. LDH has been developed as a drug delivery system because of its own perfect biosafety, biodegradability, and pH sensitivity, and the unique negative charge between layers can be exchanged with anionic drugs, peptides, and genes [73]. Mokhtari et al. [41] designed Gal-modified LDH nanocarriers for the efficient loading of Cur by nanoprecipitation (Gal-Cur/LDH). The Gal-Cur/LDH NPs could be taken up by HepG2 cells via asialoglycoprotein receptor-mediated endocytosis and exhibited selective cytotoxicity to HepG2 cells. In addition to the above-mentioned inorganic materials, gold NPs with high drug loading efficiency and good tissue penetration have also been designed to deliver Cur to play a pivotal role in tumor- or inflammation-related diseases [25,74].

As a representative of inorganic non-metallic nanomaterials, mesoporous silica NPs (MSN) have attracted more and more attention due to their high pore volume, adjustable pore size, high specific surface area, high biocompatibility, ease of surface modification, and good degradability [75]. Chen et al. [42] loaded Cur on MSN through Schiff base reaction, and modified targeting molecule FA on the surface of NPs (FA-MSN-N=C-Cur), improving the stability and bioavailability of Cur under physiological conditions (Figure 2c). The FA-MSN-N=C-Cur NPs were spherical with a uniform size distribution (Figure 2d), and the release rate of Cur was only 11.7% within 24 h under physiological conditions. Under acidic conditions, the release rate of Cur was 32.5% (pH = 6.5) or 58.6% (pH = 5.0), respectively, which can be attributed to the pH-responsiveness of the C=N bond in the Schiff base. The FA-MSN-N=C-Cur NPs exhibited a better cellular uptake ability than FA-free MSN-N=C-Cur NPs due to the overexpression of folate receptors on MCF-7 cells (Figure 2e), thus enabling targeted delivery of Cur to minimize the side effects of Cur.

### 2.5. Polymeric Nanoparticles

As a representative example, poly (lactic-co-glycolic acid) (PLGA) NPs are an extensive choice in the biomedical field, thanks to their excellent biodegradability and biocompatibility [76,77]. For example, Cur-loaded PLGA NPs were prepared using the double-emulsion solvent evaporation method, which could inhibit the migration of MDA-MB231 cells in vitro [78]. However, some urgent issues, such as partial drug loss, poor in vivo stability, slow release, and premature drug release of PLGA NPs, need to be solved so as to further enhance PLGA NPs as hydrophobic drug delivery nanoplatforms in practical clinical applications. In recent years, several studies have innovatively modulated the properties of PLGA NPs through surface modifications. Huang et al. [32] utilized the double-emulsion solvent evaporation method to synthesize Cur-loaded PLGA NPs, which were further surface modified with pluronic F127. Surface modification has solved the problems of insufficient mucus penetration and uncontrolled Cur release in the treatment of ulcerative colitis (UC). In another study, T807-modified red blood cell membrane (RBCm)-coated PLGA NPs were developed to cross the BBB for the brain delivery of Cur [33]. Polysorbate 80 coating has also been used to improve the ability of Cur-loaded PLGA NPs to cross the BBB [79].

**Figure 3:**
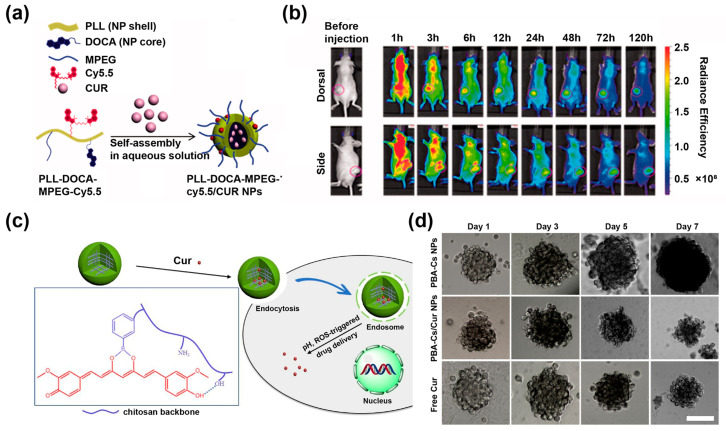
(**a**) Schematic diagram of the preparation of PLL-DOCA-MPEG-Cy5.5/Cur NPs. (**b**) In vivo whole-body near-infrared (NIR) fluorescence images of Hep3B tumor-bearing mouse models. PLL-DOCA-MPEG-Cy5.5/Cur NPs were injected via the lateral tail vein. Reproduced with permission from [43], Copyright 2018, Taylor & Francis. (**c**) Schematic illustration of the formation of PBA-Cs/Cur-NPs and pH/ROS-triggered Cur release. (**d**) Representative images of HepG2 MCTS treated with PBA-Cs NPs, PBA-Cs/Cur NPs and free Cur (Scale bar = 100 μm). Reproduced with permission from [44], Copyright 2021, Elsevier.

Compared with PLGA, the abundant amine groups on the surface of poly-L-lysine (PLL) enable it to electrostatically interact with negatively charged cell membranes for improved cellular uptake. In addition, the amine groups can be modified with targeting ligands, fluorescent molecules, and contrast agents to construct precise integrated nanoplatforms for diagnosis and treatment [43,80]. For example, PLL-DOCA-MPEG-Cy5.5/CUR NPs were prepared through the self-assembly of Cur and deoxycholic acid (DOCA)-, methoxy polyethylene glycol (MPEG)—and cyanine 5.5 (Cy5.5)-modified PLL, as shown in Figure 3a [43]. The NPs were not only effectively phagocytosed by Hep3B cells but also could stay in the body of tumor-bearing mice for 120 h (Figure 3b), suggesting the extended blood circulation time and the desired enhanced permeability and retention (EPR) effect rendered by the NPs.

Other established polymers, except PLGA and PLL, such as polylactic acid and chitosan, have also been conjugated with Cur for efficient and controlled drug delivery. Wang et al. [44] prepared phenylboronic acid-coupled chitosan (PBA-Cs-NPs) by a simple desolvation method, and successfully constructed PBA-Cs/Cur-NPs by linking PBA-Cs-NPs with Cur via boronic ester bonds (Figure 3c). The controlled release and efficient treatment of Cur were achieved based on the pH- and ROS-sensitivity of the boronic ester bonds. Therefore, compared with free Cur, PBA-Cs/Cur-NPs strongly inhibited the growth of HepG2 multicellular tumor spheroids (MCTS) within one week (Figure 3d). Other pH-responsive polymers have been used to deliver Cur via microfluidic technology to achieve tunable drug loading [81]. In addition, Pontes-Quero et al. [82] prepared Cur-loaded polymeric NPs based on α-tocopheryl methacrylate, 1-vinyl-2-pyrrolidone, and n-vinylcaprolactam, which enabled long-time drug release behavior and reduced the expression levels of inflammatory cytokines in LPS-activated macrophages. The safety and effectiveness of polymeric NPs were confirmed with the continuous improvement and progress of drug delivery and release systems of polymeric NPs, such as PLGA, PLL, and chitosan, which have greatly promoted the clinical application of polymeric NPs.

### 2.6. Other Nanoformulations

In addition to the Cur nanoformulations mentioned above, protein NPs, nanofibers, nanogels, cyclodextrin, and hollow mesoporous Prussian blue (HMPB) NPs have also been widely used for the efficient and controlled delivery of Cur. Recently, Nosrati et al. [83] prepared F@BSA@CUR NPs by encapsulating Cur in magnetic NPs coated with bovine serum albumin, and exhibited certain cytotoxicity against MCF-7 cells. Mansourizadeh et al. [84] prepared horse spleen apoferritin NPs loaded with Cur and quercetin (Que-Cur-HoS-Apo NPs) with a hydrodynamic size of 17.4 nm. The Que-Cur-HoS-Apo NPs exhibited greater cytotoxicity against MCF-7 cells than the double drug treatment alone at concentrations below 40 μM, thus triggering enhanced cell apoptosis. In another study, electrospun nanofibers of Eudragit L100 were loaded with Cur and evaluated to have fluorescence emission and antibacterial properties associated with Cur [46]. It was found that loading Cur within nanofibers not only preserved the photophysical properties of Cur but also enhanced the water solubility of Cur. Staphylococcus aureus (*S. aureus*) was completely eliminated when the released Cur concentration was 250 μg/mL. The combination of nanofibers and Cur has been developed into novel fluffy nanofiber membranes with high-efficiency air filtration and antibacterial properties for personal protection [85]. Lin et al. [86] introduced Cur into rod-shaped nanogels, which exhibited special anti-inflammatory activity in vitro and in vivo by effectively inhibiting the secretion of pro-inflammatory cytokines. Researchers have also proposed a cyclodextrin-based Cur delivery system to improve the bioavailability and antioxidant stability of Cur [87], and achieve targeted liver cancer therapy [88]. In addition, Cur, acting as a chemotherapeutic agent, can be encapsulated within HMPB NPs to achieve enhanced tumor cell apoptosis by amplifying autophagy in tumor cells [89]. Although various nanocarriers have been emerging in the design of Cur delivery, improvement of the pharmacokinetic properties of the drug, the achievement of targeted delivery to the lesion site, and the realization of microenvironment-responsive drug delivery are still key points in obtaining excellent antitumor or anti-inflammatory effects.

## 3. Cur Nanoformulations for Inflammatory Diseases

Excessive and persistent inflammatory responses can lead to tissue lesions and dysfunction, and even evolve into arthritis, neurological diseases, pneumonia, etc. The nanomaterials discussed above have been successfully used to enhance the biological function of Cur and to improve its therapeutic efficacy. Therefore, the development of promising Cur nanoformulations is imperative for the treatment of inflammatory diseases. This section mainly summarizes the treatment of different Cur nanoformulations in various inflammatory diseases (as shown in Table 2).

### 3.1. Cur Nanoformulations for Arthritis Management

RA, gouty arthritis, and osteoarthritis are all autoimmune diseases characterized by joint inflammation. Clinical symptoms include cartilage and bone damage, osteoporosis, and deterioration of joint function, which can lead to disability in severe cases [97]. As an extracellular matrix, hyaluronic acid (HA) has good biological lubricating effects, which can protect the articular surface of RA patients from erosion and reduce adverse reactions caused by drugs. Therefore, micelles composed of HA and Cur (HA-Cur) were synthesized for the combined treatment of anti-inflammatory and anti-joint wear in RA [91]. The ability of HA-Cur micelles to downregulate the levels of inflammatory cytokines (TNF-α and IL-1) was better than that of free Cur, indicating that HA-Cur micelles improve the bioavailability and amplify the biological function of Cur. In the mouse RA model, the poor water solubility of Cur increased the friction of the cartilage at the joint site, while the lubricating effect of HA made the surface of the articular cartilage smooth, and foot edema disappeared in the treatment group HA-Cur micelles.

**Figure 4:**
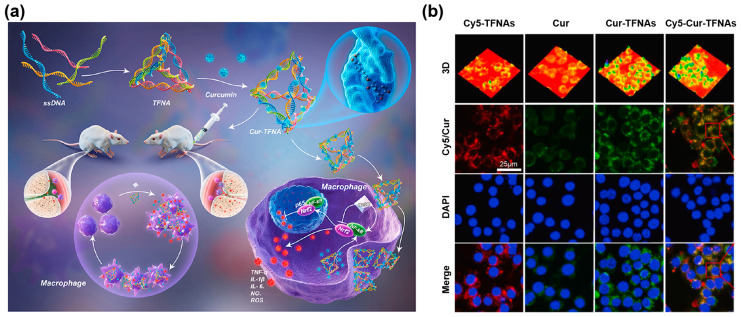
(**a**) Schematic illustration of acute gouty arthritis treatment with Cur-TFNAs. (**b**) Cellular uptake of Cy5-TFNAs, Cur, Cur-TFNAs, or Cy5-Cur-TFNAs in RAW264.7 cells. Reproduced with permission from [90], Copyright 2022, KeAi.

Other Cur nanoformulations focused on regulating inflammation-related cells and their cellular signaling pathways have also been developed. Zhang et al. [90] utilized tetrahedral framework nucleic acids (TFNAs) with unparalleled advantages in biocompatibility, structural stability, and programmability as carriers for the delivery of Cur (Cur-TFNAs) (Figure 4a). As displayed in Figure 4b, the phagocytosis of Cur-TFNAs by RAW264.7 cells was about 6 times higher than that of free Cur. The treatment of Cur-TFNAs successfully down-regulated the secretion of TNF-α, IL-6, and iNOS in LPS-activated macrophages by regulating the NF-κB signaling pathway and activating the nuclear factor E2-related factor (Nrf)-2 signaling pathway to guide the expression of antioxidant enzymes, such as superoxide dismutase (SOD), heme oxygenase-1 (HO-1), and Nrf-2, to protect macrophages from oxidative stress damage.

Besides the elimination of inflammatory factors to relieve joint swelling and pain, inhibition of chondrocyte apoptosis to reverse cartilage damage is also a promising strategy for treating arthritis. Iron-Cur-based coordination NPs (Fe-Cur NPs) have been developed to effectively scavenge intracellular ROS in an IL-1β-induced chondrocytes to inhibit oxidative stress-induced chondrocyte death [92]. The results of safranin O/fast green staining suggested poor treatment of Cur, showing that there still remains proteoglycan loss and cartilage damage in osteoarthritis mice. In contrast, osteoarthritis mice treated with Fe-Cur NPs had smooth articular surfaces and regenerated cartilage tissue, which benefited from the enhanced bioavailability of Cur by Fe-Cur NPs and activated the Nrf-2 signaling pathway to inhibit the upregulation of the nodular receptor protein 3 (NLRP3) inflammasome in activated chondrocytes. Therefore, compared with conventional chemotherapy, the adoption of Cur nanoformulations enhances the therapeutic effect of arthritis through sustained drug release and increased drug concentration in the lesion area.

### 3.2. Cur Nanoformulations for Neurodegenerative Disease Management

Common neurodegenerative diseases include Alzheimer’s disease (AD), Parkinson’s disease (PD), and brain damage. Among the various small molecular drugs used for the treatment of neurodegenerative diseases, their limited BBB permeability is often compensated for by increasing administration frequency, thereby aggravating the adverse effects of the drugs and limiting their clinical therapeutic effects [98]. However, nanomedicines with targeted and sustained drug-release properties have been an emerging strategy for crossing the BBB. Removing amyloid-β (Aβ) deposits, inhibiting the phosphorylation of Tau protein, and relieving oxidative stress to protect neuronal cells are the targets of these nanomedicines for the treatment of AD [99]. In a recent study, an NIR stimuli-responsive delivery system based on polymer-dispersed liquid crystal (PDLC) NPs was designed by integrating high Aβ-binding affinity, stimuli-responsive drug release, and photothermal degradation properties for efficient detection and photodegradation of Aβ [100]. Benefiting from the excellent Aβ peptide binding ability of the system, the PDLC NPs could effectively release the encapsulated Cur under NIR laser irradiation, and synergized with the Leu-Pro-Phe-Phe-Asp (LPFFD) peptide anchored on the surface of PDLC NPs to mitigate the aggregation degree of Aβ by 77%, which improved the anti-amyloid ability of Cur. Despite the fact that numerous studies have suggested that clearing Aβ deposits is a promising therapeutic strategy, nearly all Aβ-targeted drug candidates have failed in clinical trials.

**Figure 5:**
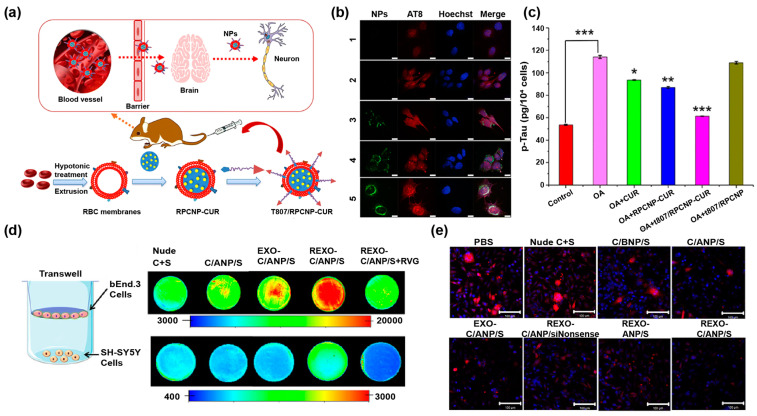
(**a**) Schematic illustration of the preparation of T807/RPCNP-CUR NPs and their application for AD. (**b**) CLSM images of okadaic acid (OA)-treated HT22 cells treated with different NPs after 4 h incubation (Nuclei were stained with blue fluorescence; p-Tau was shown in red fluorescence; COU-labeled PCNP, RPCNP, and T807/RPCNP were shown in green fluorescence. 1: control; 2: OA; 3: OA + PCNP-COU6; 4: OA + RPCNP-COU6; 5: OA + T807/RPCNP-COU6. Scale bars = 100 μm). (**c**) Elisa analysis of cellular total p-Tau levels after treatment with different NPs in OA-treated HT22 cells. Here, *** indicates *p* < 0.001, ** indicates *p* < 0.01, and * indicates *p* < 0.05, respectively (compared to the OA group). Reproduced with permission from [33], Copyright 2020, Springer Nature. (**d**) Scheme of the Transwell system and the internalization of different nanomaterials in bEnd.3 cells (top) and SH-SH5Y cells (bottom). (**e**) The expression of α-syn in SNCA–mCherry–SH-SY5Y cells after treatment with different NPs (Scale bars = 100 μm). Reproduced with permission from [105], Copyright 2020, AAAS.

As a Tau pathology that can occur independently from Aβ, phosphorylation of Tau leads to the development of neurofibrillary tangles, triggering neuronal cell death. Therefore, phosphorylated tau (p-Tau) has become a new target for treating AD [101]. So far, several Tau-targeted Cur nanoformulations have been constructed for the treatment of AD. In a recent study, RBCm-coated PLGA NPs bearing T807 molecules were designed to load Cur (T807/RPCNP-CUR, Figure 5a) [33]. As shown in Figure 5b,c, the developed T807/RPCNP-CUR could target p-Tau and inhibit the expression of p-Tau in HT22 cells to almost normal levels, indicating that the nanoplatform could be used for targeted degradation of p-Tau to relieve AD symptoms. In another study, Cur was loaded into RBCm-camouflaged human serum albumin NPs (Cur-loaded T807/TPP-RBC), and the AD therapeutic targets were localized into neuronal cell mitochondria, as guided by T807 and triphenylphosphine (TPP) molecules carried by the RBCm [93]. Cur-loaded T807/TPP-RBC NPs could successfully cross the BBB to exert the antioxidant effect of Cur, leading to reduced mitochondrial oxidative stress and decreased neuronal death for AD alleviation. Notably, the differences in bioavailability, drug release behavior, and brain tissue distribution associated with various Cur nanoformulations endow them with different potentials to alleviate neuroinflammation [102].

In addition to the therapeutic application of Cur nanoformulations in AD, the existing literature supports Cur as a small molecular drug that reduces α-synuclein (α-syn) aggregates in the treatment of PD [103,104]. However, its low bioavailability and difficulty in crossing the BBB to accumulate at neuronal sites are the primary issues to be addressed in the development of Cur therapeutic strategies for PD. Therefore, Liu et al. [105] prepared an engineered core-shell hybrid system based on rabies virus glycoprotein peptide (RVG)–modified exosome (EXO) Cur/phenylboronic acid-poly(2-(dimethylamino)ethyl acrylate) nanoparticle/small interfering RNA targeting SNCA (REXO-C/ANP/S) for the treatment of PD. In an in vitro BBB model, REXO-C/ANP/S NPs smoothly passed the barrier formed by the upper epithelial cells (bEnd.3 cells) and entered SH-SY5Y cells in the lower chamber, while the ability to cross the BBB in vitro was blocked by free RVG treatment (Figure 5d). Furthermore, REXO-C/ANP/S NPs, acting as a nano-scavenger, showed less α-syn expression level (red fluorescence) than other groups (Figure 5e), suggesting the superior ability of REXO-C/ANP/S NPs to clear α-syn aggregates for efficient AD therapy.

In addition to the modification of cell-penetrating peptides and cell-targeting molecules to enhance Cur delivery in the brain, physically disrupting the BBB can also achieve the same goal. Zhang et al. [94] synthesized Cur-loaded polybehenate 80-modified cerasome (CPC NPs), and combined it with ultrasound-targeted microbubble destruction (UTMD) technology to open the BBB, thus enhancing the local delivery of Cur to the brain. The treatment resulted in Cur-mediated depletion of α-syn and restored the motor behavior and dopamine (DA) levels of the PD mice to normal. Taking anti-oxidation, anti-inflammatory, and inhibition of neuronal cell death as the starting point, Cur nanoformulations have also been developed for the treatment of traumatic brain injury (TBI), and can effectively relieve neuroinflammation, reduce brain edema and brain tissue loss, and improve TBI mice cognitive function [95,106].

### 3.3. Cur Nanoformulations for Respiratory Disease Management

Cytokine storm is a life-threatening systemic inflammatory syndrome involving elevated levels of cytokines and hyperactivation of immune cells, leading to secondary organ dysfunction, particularly in the kidney, liver, or lung [107]. The cytokine storm is a common physiological and pathological event in severe coronavirus disease-19 (COVID-19) patients, and inhibition of the cytokine storm is helpful in the treatment of COVID-19. For instance, Sharma et al. [108] synthesized Cur-encapsulated polysaccharide NPs (CUR-PS-NPs) that could inhibit SARS-CoV-2 spike protein (CoV2-SP)-induced cytokine storms in Huh7.5 liver epithelial cells and A549 lung epithelial cells by inhibiting the NF-κB signaling pathway and by inactivating the MAPK signaling pathway.

**Figure 6:**
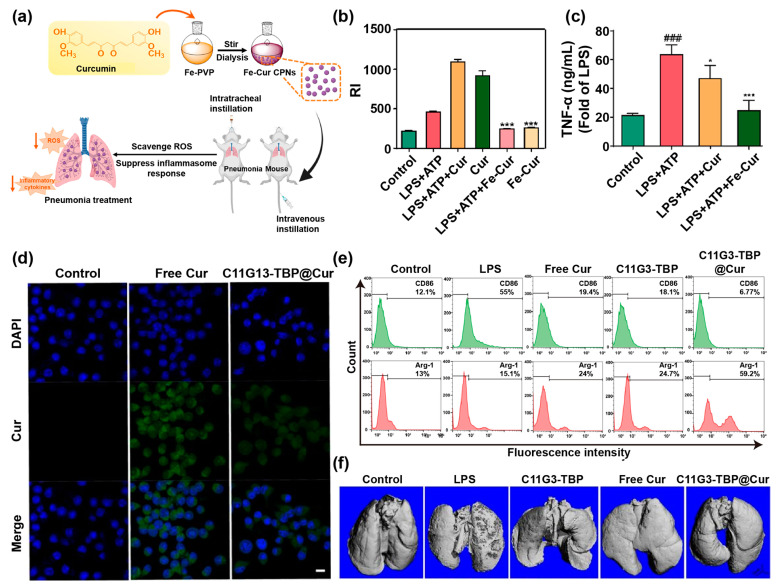
(**a**) Schematic illustration of Fe-Cur NP synthesis and its therapeutic treatment in acute lung injury. (**b**) Quantitative results of the fluorescence intensity of ROS in J774A.1 cells. (**c**) The content of TNF-α in J774A.1 cells treated with different NPs. The ### indicates *p* < 0.001 as compared with the control group, while the * indicates *p* < 0.05, and *** indicates *p* < 0.001 compared with the LPS + ATP group. Reproduced with permission [109], Copyright 2022, American Chemical Society. (**d**) CLSM images of LPS-activated MH-S cells treated with PBS, Cur, and C11G3-TBP@Cur for 4 h (Scale bar = 10 μm). (**e**) Detection of CD86 and Arg-1 expression in MHS cells treated with different NPs by flow cytometry. (**f**) Micro-CT images of lung tissue in different treatment groups. Reproduced with permission [22], Copyright 2022, Ivyspring.

In addition to suppressing cytokine storms, combined ROS scavenging strategies have become a broad choice for the treatment of respiratory inflammation. In a recent example, Yuan et al. [109] reported metal coordination-based iron-Cur NPs with nanozyme functionalities for the treatment of acute lung injury (Fe-Cur NPs, Figure 6a). As shown in Figure 6b, the Fe-Cur NPs significantly reduced the ROS content in the J774A cells. Moreover, the Fe-Cur NPs down-regulated pro-inflammatory cytokines (TNF-α) compared to treatment with free Cur, as the Fe-Cur NPs enhanced the bioavailability of Cur and its accumulation in cells (Figure 6c). Li et al. [22] encapsulated Cur in phosphorus dendron micelles (C11G3-TBP) with anti-inflammatory activity by self-assembly to construct C11G3-TBP@Cur for the combined treatment of acute lung injury. As can be seen in Figure 6d, LPS-activated MHS cells could successfully take up the C11G3-TBP@Cur complexes. The C11G3-TBP@Cur inhibited the expression of the M1 macrophage marker “cluster of differentiation 86” (CD86) (from 55% to 6.77%) and increased the level of the M2 macrophage marker “arginase-1” (Arg-1) (from 15.1% to 59.2%), showing that C11G3-TBP@Cur had a stronger anti-inflammatory activity than the drug-free micelles and free Cur (Figure 6e). The Micro-CT imaging of lung tissue showed that the surface of the lung tissue after C11G3-TBP@Cur treatment was smooth, and the treatment effect was better than C11G3-TBP and free Cur (Figure 6f). Collectively, Cur nanoformulations may serve as a reasonable platform for the treatment of respiratory diseases.

### 3.4. Cur Nanoformulations for Ulcerative Colitis Management

**Figure 7:**
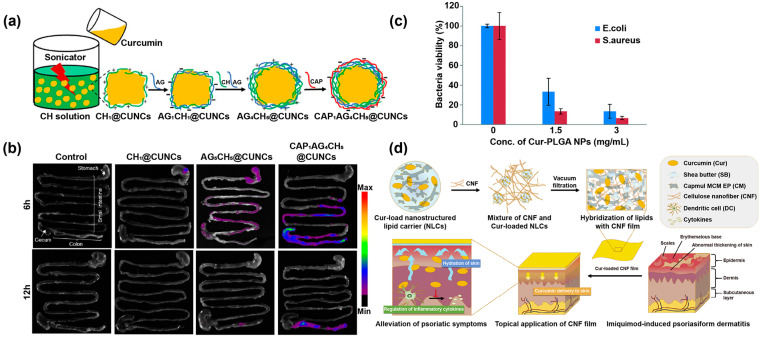
(**a**) Schematic diagram of the preparation of CAP_1_AG_4_CH_5_@CUNC. (**b**) The distribution of different NPs in the gastrointestinal tract of mice after 6 h or 12 h administration. Reproduced with permission [111], Copyright 2020, American Chemical Society. (**c**) Antimicrobial effect of Cur-PLGA NPs against *E. coli* and *S. aureus*. Reproduced with permission [112], Copyright 2019, Wiley-VCH. (**d**) Schematic illustration of the Cur-loaded lipid-hybridized CNF film for the treatment of IMQ-induced psoriasiform dermatitis. Reproduced with permission [113], Copyright 2018, Elsevier.

The inflammatory response in ulcerative colitis (UC) mainly persistently affects the colon and rectum [110], and the development of Cur nanoformulations for colon-targeted drug delivery becomes a challenging task due to the special location of the colon. Oshi et al. [111] prepared Cur nanocrystals (CUNCs) surrounded by chitosan (CH), sodium alginate (AG), and cellulose acetate phthalate (CAP) polyelectrolyte multilayers as core−shell NPs for oral administration to treat UC (Figure 7a). The Cur release rate from the core-shell NPs was lower at pH values that mimicked the stomach and small intestine, whereas the rate of Cur release was higher at pH values that mimicked the colon. Furthermore, mouse gastrointestinal biodistribution studies showed that the distribution of CAP1AG4CH5@CUNGs in the colon was significantly higher than in the stomach and small intestine at 6 h and 12 h, respectively (Figure 7b). Overall, the designed CAP1AG4CH5@CUNGs were efficiently enriched in the colon site, which greatly improved the treatment efficiency of UC.

In order to solve the problem of the reduced therapeutic effect of UC due to the insufficient mucus penetration ability of Cur, Huang et al. [32] encapsulated Cur and catalase in PLGA NPs (CUR/CAT-NPs), and introduced PF127 on the surface of CUR/CAT-NPs (P-CUR/CAT-NPs) to facilitate their mucus penetration. The P-CUR/CAT-NPs can effectively relieve UC symptoms by scavenging ROS in LPS-activated RAW264.7 cells and reducing inflammatory cytokines in the serum of UC mice. Although skillfully designed Cur nanoformulations can effectively accumulate in the colons of UC patients, their poor stability in the digestive tract and short residence time in the colon are other important issues to decrease the UC treatment efficacy of Cur nanoformulations. For this reason, Luo et al. [45] fabricated an oral food-grade nanocarrier composed of tannic acid (TA)-coated, genipin (Gnp)-crosslinked human serum albumin (HSA) to encapsulate Cur (TA/Cur-NPs). The TA-Cur-NPs improved the drawbacks of the burst release of Cur, and the cumulative Cur release amount reached 60% within 100 h, indicating the good sustained release performance of the TA-Cur-NPs. The employed TA coating effectively improved the adhesion of TA-Cur-NPs to the inflammatory mucosa and prolonged the retention time of TA-Cur-NPs in colon tissue. The TA-Cur-NPs downregulated the expression of inflammatory factors, such as myeloperoxidase (MPO), IL-6, TNF-α, and iNOS, in colon tissue by inhibiting the toll-like receptor 4 (TLR4) signaling pathway.

In order to improve the ability of Cur nanoformulations to be specifically internalized by cells when administered orally or intravenously and to exhibit the function of releasing Cur on demand, Gou et al. [114] prepared natural silk fibroin (SF) NPs with chondroitin sulfate (CS) functionalized on the surface and Cur encapsulated inside (CS-Cur NPs). The cellular internalization efficiency of CS-Cur NPs was significantly reduced in the presence of free CS molecules, indicating that CS-Cur-NPs could be efficiently taken up by macrophages through CD44 receptor-mediated endocytosis. The CS-Cur-NPs with targeting function obviously inhibited the expression of inflammatory factors, and the therapeutic effect of intravenous administration was better than that of oral administration. These findings indicate the promising therapeutic potential of Cur nanoformulations in the treatment of UC.

### 3.5. Cur Nanoformulations for Other Inflammatory Disease Management

Cur nanoformulations have also been successfully used for antibacterial, wound healing, antipsoriasis, and other inflammatory diseases. For example, Liu et al. [112] synthesized Cur-loaded PLGA NPs (Cur-PLGA NPs) via an emulsion-solvent evaporation method to inhibit the viability of Escherichia coli (*E. coil*) and *S. aureus* to less than 50% at a concentration of 1.5 mg/mL (Figure 7c). Zou et al. [96] prepared Cur-loaded covalent organic frameworks (Cur@COF) by a one-pot method, and further introduced Cur@COF into polycaprolactone (PCL) nanofibrous membranes by electrospinning (Cur@COF/PCL NFMs) for antimicrobial and wound healing therapy. The Cur@COF/PCL NFMs inhibited *E. coli* and *S. aureus* by more than 60% when the Cur content was 10%, thus reducing the risk of bacterial infection during the treatment. In addition, the Cur@COF/PCL NFMs significantly reduced the expression level of TNF-α in the wound tissue, maintained the wound shrinkage rate to be 98.17%, and almost completely healed the wound on the 14th day. Kang et al. [113] hybridized Cur-loaded nanostructured lipid carriers (NLCs) in cellulose nanofibers (CNF) to construct a mixture of CNF and Cur-loaded NLCs, and the Cur-NLCs-CNF films obtained after vacuum filtration were used as a topical drug delivery system in the treatment of psoriasis (Figure 7d). In the imiquimod (IMQ)-induced psoriatic mice model, the Cur-NLCs-CNF films reduced pro-inflammatory cytokine levels in the skin and ameliorated psoriatic skin symptoms. Since the body’s inherent skin barrier limits the therapeutic efficacy and bioavailability of Cur nanoformulations, some studies have shown that Cur liposomes modified with TD polypeptide (ACSSSPSKHCG) can improve the transdermal efficiency of Cur for efficient topical antipsoriasis treatment [115]. In conclusion, the Cur nano-delivery system is expected to be developed for the treatment of more inflammatory diseases and to continuously promote the application of Cur in clinical medicine.

## 4. Conclusions and Future Perspectives

Based on the multiple biological functions of Cur, some drugs containing Cur ingredients have been approved to treat certain diseases; for example, *Jiangzhi Tongluo Soft Capsules* containing 50 mg of Cur-like compounds per capsule have been approved for the treatment of hyperlipidemia. Nevertheless, Cur with anti-inflammatory, antioxidant, and antibacterial properties, has difficulties in achieving the desired therapeutic effect due to its poor water solubility and low bioavailability. To overcome these obstacles, intelligent nanocarriers have been designed to combine with Cur to expand the diverse functions of Cur, such as high bioavailability, good drug release behavior, prolonged in vivo circulation time, and targeted therapy. We review the different types of Cur nanoformulations, including liposomes, polymeric micelles, MOFs, inorganic nanocarriers, polymeric NPs, and nanofibers, and discuss the features of each carrier system are discussed. The recent progress of the designed Cur nanoformulations to treat various inflammatory diseases, such as arthritis, neurological diseases, respiratory diseases, UC, and psoriasis, is also discussed, thus providing new options and insights to address the limitations of Cur in clinical applications.

The safety and therapeutic efficacy of Cur nanoformulations are important factors being considered in clinical trials. Related studies have shown that short-term intravenous administration of liposomal Cur at 120 mg/m^2^ was safe (NCT01403545) [116]. In another randomized double-blind placebo-controlled clinical trial, 1% nanocurcumin gel combined with 0.1% triamcinolone acetonide was shown to be an effective treatment strategy to improve the efficacy of oral lichen planus compared with triamcinolone acetonide alone (IRCT20190523043678N1) [117]. In addition, Cur nanomicelles can reduce levels of IL-6, TNF-α and monocyte chemoattractant protein-1 in the serum of patients, suggesting the feasibility and potential clinical benefit of eliminating inflammation in hemodialysis patients and treating migraine (IRCT20180328039154N1, IRCT20160626028637N2) [118,119].

Despite the advantages of Cur nanoformulations for the treatment of inflammatory diseases in preclinical studies, they still have many limitations in clinical trials. For example, the preparation process of most Cur nanoformulations is complex, which makes it difficult to achieve large-scale production and accurate quality control, and there is a lack of understanding of the long-term biosafety of Cur nanoformulations. These are the great challenges in the process of transforming Cur nanoformulations from the laboratory stage to clinical applications. Future research on Cur nanoformulations should be carried out to address these challenges. In addition, nanocarriers with other properties and functions can be integrated into host nanomaterials to prepare multifunctional Cur delivery systems. Further, it is also necessary to combine Cur with other drugs, antibodies, or active ingredients, such as enzymes and proteins, in order to obtain excellent precise targeted combination therapy effects. Lastly, strategies to tailor the corresponding Cur nanoformulations to different pathological mechanisms also need to be developed to achieve individualized treatments.

## Figures and Tables

**Table 1:** Representative types, compositions, physical properties, and functionalization modifications of various Cur nanoformulations.

Formulation	Composition	Modification	Size/PDI	Zeta Potential	Encapsulation Efficiency	Activity	Refs.
Liposomes	Soybean phospholipid (SPC), hydrogenated soybean phospholipid (HSPC), and cholesterol	–	183 nm/0.37	−1.4 mV	87.1%	Improving the stability and release performances of Cur	[35]
	Soybean lecithin and cholesterol	Creating an acidic microenvironment in liposomes	325 nm/0.27	−18.6 mV	73.7%	Improving the encapsulation efficiency and physical stability of Cur	[36]
	L-α-phosphatidylcholine and cholesterol	Glycyrrhetinic acid (GA), galactose (Gal)	139 nm/0.17	−38.8 mV	88.0%	Endowing Cur with targeting properties	[30]
Polymeric micelles	Polyethyleneoxide100-polypropyleneoxide65-polyethyleneoxide100 (PEO100-PP065-PEO100)	Silicone hybrid	14 nm/–	−6.5 mV	–	Enhancing the chemotherapeutic effect of Cur	[37]
	Phosphorus dendron	–	114 nm/–	−50 mV	96.8%	Synergizing Cur with phosphorus dendron micelles to exert anti-inflammatory effect	[22]
MOFs	2-methylimidazole and Zn^2+^	–	119 nm/–	4.3 mV	88.2%	High encapsulation efficiency and controlled Cur release	[38]
		Folic acid (FA)	200 nm/0.12	−8.6 mV	–	Targeting cancer cells for controlled Cur release	[39]
	1,3,5-Benzenetricarboxylic acid and Fe^3+^	Polydopamine-modified hyaluronic acid	200 nm/–	−18 mV	94.3%	Strong photothermal conversion efficiency, good dispersion and stability	[40]
Inorganic nanocarriers	Layered double hydroxide (LDH)	Galactose	116 nm/–	10 mV	–	High endocytosis efficiency and pH responsiveness	[41]
	Mesoporous silica NPs (MSN)	FA	165 nm/–	−8.9 mV	49.6%	FA-targeted and controlled release drugs	[42]
Polymeric NPs	Poly (lactic-co-glycolic acid) (PLGA)	Pluronic F127	274 nm/0.10	−14 mV	51.4%	Improving Cur bioavailability and promoting mucosal penetration	[32]
		T807 functionalized red blood cell membrane (RBCm)	170 nm/0.10	8.9 mV	–	Efficiently penetrate the blood–brain barrier and inhibit neuronal cell death by binding to phosphorylated tau (p-tau) protein	[33]
	Poly-L-lysine (PLL)	Deoxycholic acid (DOCA), methoxy polyethylene glycol (mPEG) and cyanine 5.5 (Cy5.5)	246 nm/–	–	78.5%	Enhancing the encapsulation rate of Cur and prolonging the blood circulation time of Cur	[43]
	Chitosan	Phenylboronic acid	244 nm/0.2	–	83.5%	High Cur loading rate and pH, ROS-triggered Cur release behavior	[44]
Proteins	Genipin-crosslinked human serum albumin	Tannic acid (TA)	220 nm/0.2	−28.8 mV	86.0%	Prolonging the colonic adhesion time of Cur	[45]
Nanofibers	Eudragit L100 fibers	–	–	–	–	Overcoming the low water solubility of Cur	[46]

“–” represents “not available”.

**Table 2:** Representative application of Cur nanoformulations in inflammatory diseases.

Disease	Nanoformulation	Cell	Therapeutic Target	Refs.
Acute gouty arthritis	Tetrahedral framework nucleic acids	RAW 264.7	Regulating the NF-κB signaling pathway, and activating nuclear factor E2-related factor-2 signaling pathway	[90]
RA	Micelles	–	Anti-inflammatory and anti-joint wear	[91]
Osteoarthritis	Iron-Cur-based coordination NPs	Chondrocytes	Inhibiting chondrocyte apoptosis by inhibit the activation of the nodular receptor protein 3 (NLRP3) inflammasome	[92]
Alzheimer’s diseases	PLGA NPs	HT22	Targeting p-Tau and inhibiting the expression of p-Tau	[33]
	Human serum albumin	HT22	Reduced mitochondrial oxidative stress and decreased neuronal death	[93]
Parkinson’s diseases	Cerasomes	SH-SY5Y	Reducing expression of α-syn	[94]
Traumatic brain injury	Hydrogels	–	Antioxidation, anti-inflammatory and inhibition of neuronal cell death	[95]
Acute lung injury	Micelles	MHS	Scavenging ROS and inhibiting the nuclear translocation of NF-κB	[22]
Ulcerative colitis	PLGA NPs	RAW 264.7	Scavenging ROS and reducing inflammatory cytokines	[32]
	Human serum albumin	Caco-2	Downregulating the expression of inflammatory factors by inhibiting the toll-like receptor 4 (TLR4) signal-ing pathway	[45]
Wound	Nanofibrous membranes	–	Accelerating wound healing and skin regeneration by reducing the expression of inflammatory factors and enhancing the expression of angiogenesis	[96]

“–” represents “Not reported”.

## Data Availability

Not applicable.
